# Supplementary material for: The Presence of Four Pathogenic Oral Bacterial Species in Six Wild Snake Species from Southern Taiwan: Associated Factors
Source: Microorganisms. 2024 Jan 26;12(2):263. doi: 10.3390/microorganisms12020263 (PMC10891919; doi:10.3390/microorganisms12020263)
Supplement: Supplementary file 1 [file microorganisms-12-00263-s001.zip › microorganisms-2825123-supplementary.pdf]

Supplementary Materials:

Table S1. Microorganisms previously documented in the literature as associated with secondary infections resulting from snake bites [8,23,44,46,73,80-83].

Aerobic Bacteria		Anaerobic Bacteria
Gram-positive	Gram-negative	
Bacillus subtilis	*Aeromonas hydrophila*	*Bacteroides fragilis*
Bacillus megaterium	*Bordetella* species	*Clostridium perfringens*
Bacillus thuringiensis	*Citrobacter freundii*	*Clostridium sporogenes*
Bacillus cereus	*Citrobacter amalonaticus*	*Cetobacterium* species
Corynebacterium diphtheriae	*Chryseobacterium* species	*Clostridium* species
Enterococcus species	*Escherichia coli*	*Peptostreptococcus* species
Lactococcus lactis	*Enterobacter aerogenes*	
Micrococcus luteus	*Klebsiella pneumoniae*	
Micrococcus glutamicus	*Morganella morganii*	
Rhodococcus species	*Proteus vulgaris*	
Staphylococcus aureus	*Proteus mirabilis*	
Strptococcus species	*Providencia rettgeri*	
Vagococcus species	*Pseudomonas aeruginosa*	
	Salmonella typhi	
	Serratia marcescens	
	Serratia liquefaciens	
	Salmonella species	
	Stenotrophomonas species	
	Shewanella species	
	Vibrio cholera	
	Yersinia enterocolitica	
